# Supplementary material for: The causal relationship between the human gut microbiota and pyogenic arthritis: a Mendelian randomization study
Source: Front Cell Infect Microbiol. 2024 Nov 26;14:1452480. doi: 10.3389/fcimb.2024.1452480 (PMC11629706; doi:10.3389/fcimb.2024.1452480)
Supplement: Supplementary file 1 [file DataSheet1.zip › mendelian test/ebi-a-GCST90027796.csv_leave_one_out.pdf]

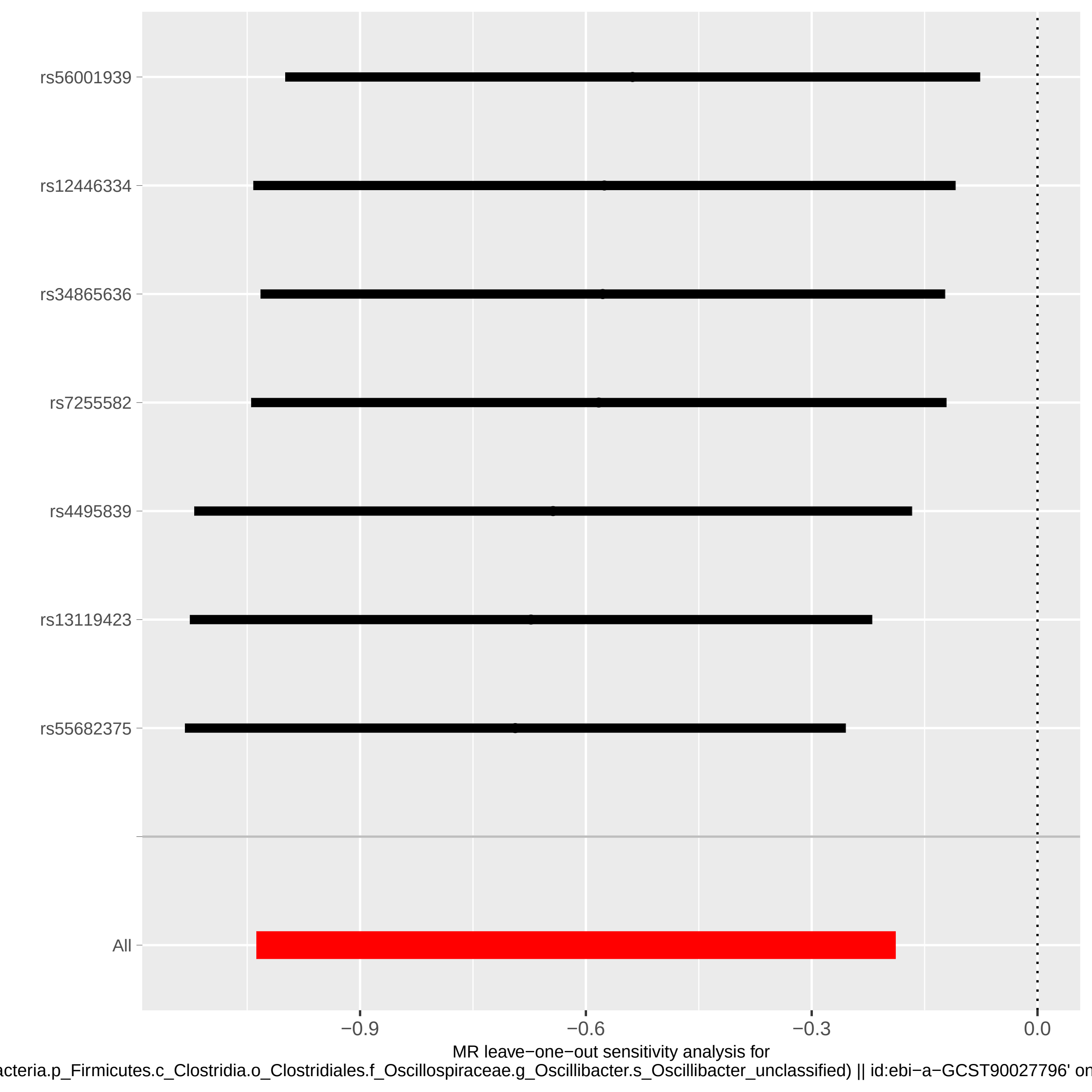
rs56001939
rs12446334
rs34865636
rs7255582
rs4495839
rs13119423
rs55682375
All
−0.9
−0.6
−0.3
0.0
MR leave−one−out sensitivity analysis for
acteria.p_Firmicutes.c_Clostridia.o_Clostridiales.f_Oscillospiraceae.g_Oscillibacter.s_Oscillibacter_unclassified) || id:ebi−a−GCST90027796' on
